# Supplementary material for: Co-Inhibition of PARP and STAT3 as a Promising Approach for Triple-Negative Breast Cancer
Source: Biomolecules. 2025 Jul 17;15(7):1035. doi: 10.3390/biom15071035 (PMC12292728; doi:10.3390/biom15071035)
Supplement: Supplementary file 1 [file biomolecules-15-01035-s001.zip › biomolecules-3598291-supplementary.pdf]

Supplemental Information

Co-Inhibition of PARP and STAT3 as a Promising Approach for Triple-Negative Breast Cancer

Figure S1

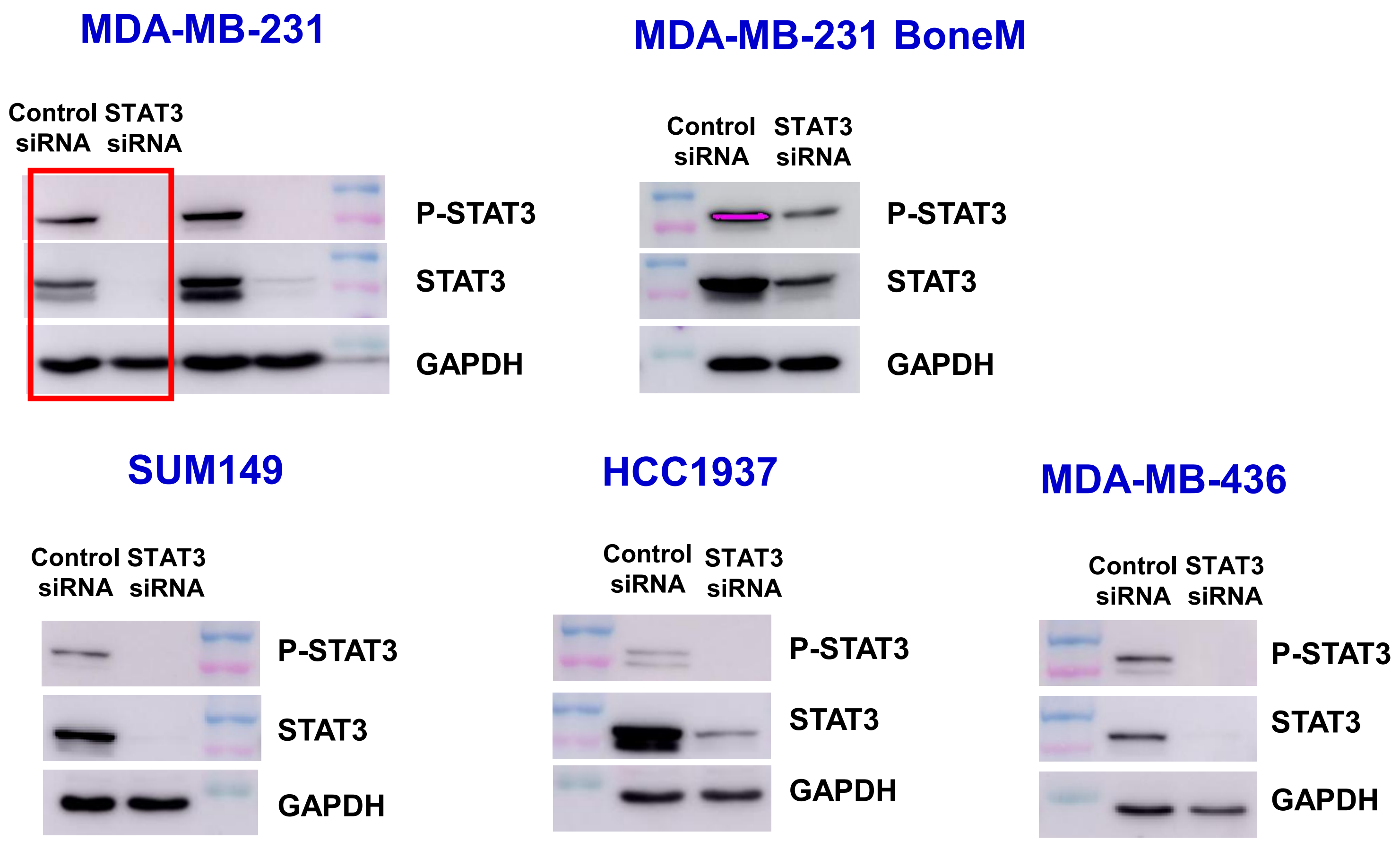

Western blot showing all bands with molecular weight markers for Figure 2A.

Figure S2

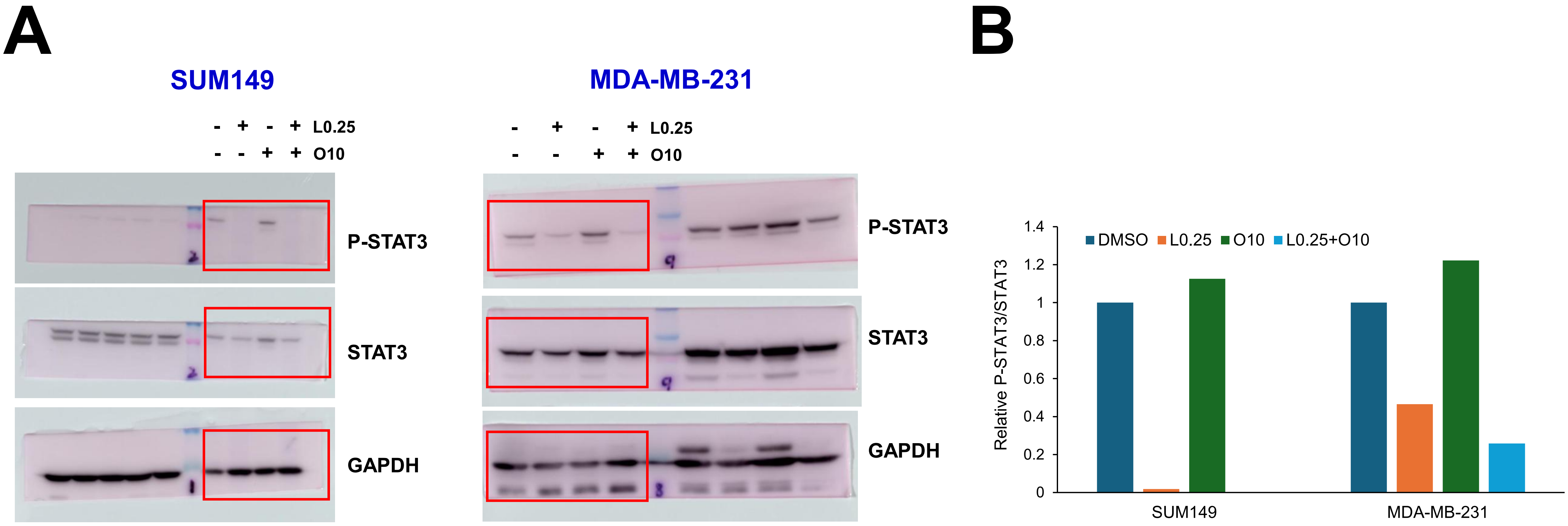

(**A**) Western blot showing all bands with molecular weight for Figure 4A. (**B**) Quantification of phosphorylated STAT3 (P-STAT3) levels normalized to total STAT3 for Figure 4A.
